# Pipeline for the removal of hardware related artifacts and background noise for Raman spectroscopy

**DOI:** 10.1016/j.mex.2020.100883

**Published:** 2020-04-21

**Authors:** Christian J.F. Bertens, Shuo Zhang, Roel J. Erckens, Frank J.H.M. van den Biggelaar, Tos T.J.M. Berendschot, Carroll A.B. Webers, Rudy M.M.A. Nuijts, Marlies Gijs

**Affiliations:** a University Eye Clinic Maastricht, Maastricht University Medical Center+, P. Debyelaan 25, P.O. Box 5800, 6202 AZ Maastricht, Netherlands; b Maastricht University, School for Mental Health and Neuroscience, University Eye Clinic Maastricht, Universiteitssingel 50, P.O. Box 616, 6200 MD Maastricht, Netherlands; c Chemelot Institute for Science and Technology (InSciTe), Gaetano Martinolaan 63-65, 6229 GS Maastricht, Netherlands

**Keywords:** Raman spectroscopy, Ketorolac tromethamine, Data processing, Rabbits, Ophthalmology

## Abstract

Raman spectroscopy is a real-time, non-contact, and non-destructive technique able to obtain information about the composition of materials, chemicals, and mixtures. It uses the energy transfer properties of molecules to detect the composition of matter. Raman spectroscopy is mainly used in the chemical field because background fluorescence and instrumental noise affect biological (*in vitro* and *in vivo*) measurements. In this method, we describe how hardware related artifacts and fluorescence background can be corrected without affecting signal of the measurement. First, we applied manual correction for cosmic ray spikes, followed by automated correction to reduce fluorescence and hardware related artifacts based on a partial 5^th^ degree polynomial fitting and Tophat correction. Along with this manuscript we provide a MatLab^Ⓡ^ script for the automated correction of Raman spectra.

• “*Polynomial_Tophat_background_subtraction _methods.m*” offers an automated method for the removal of hardware related artifacts and fluorescence signals in Raman spectra.
• “*Polynomial_Tophat_background_subtraction _methods.m*” provides a modifiable MatLab file adjustable for multipurpose spectroscopy analysis.
• We offer a standardized method for Raman spectra processing suitable for biological and chemical applications for modular confocal Raman spectroscopes.

## Graphical abstract

**Figure:**
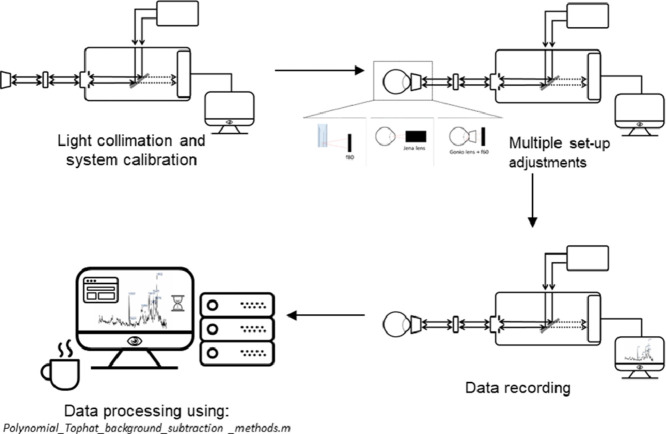

## Specifications table

**Table:** 

Subject Area:	Pharmacology, Toxicology and Pharmaceutical Science
More specific subject area:	A new method combining background fluorescence, and instrumental artifact reduction based on Top-hat filtering for Raman spectroscopy.
Method name:	Partial 5^th^ degree polynomial fitting, and Tophat filtering according to: Perez-Pueyo, R., M.J. Soneira, and S. Ruiz-Moreno, Morphology-based automated baseline removal for Raman spectra of artistic pigments. Appl Spectrosc, 2010. 64(6): p. 595–600.
Name and reference of original method:	Bertens, C.J.F., et al., *Confocal Raman spectroscopy: Evaluation of a non-invasive technique for the detection of topically applied ketorolac tromethamine in vitro and in vivo.* Int J Pharm, 2019. 570: p. 118,641. Doi:10.1016/j.ijpharm.2019.118641
Resource availability:	Software:
	• OriginPro 9.0.0 (64 bit ed. OriginLab corp. Northampton, US)
	• MatLab^Ⓒ^ (Version 2017b, The Mathworks Inc., Natick, MA, US)
	See: *2.1 Materials* for more details

## Introduction

Raman spectroscopy is a vibrational spectroscopic technique, based on an energy transfer between an illuminated sample and the irradiated light. In contrast with *e.g.* infrared (IR) spectroscopy, which analyses absorbed and transmitted fractions of the light, Raman spectroscopy makes use of scattered radiation. Although the predominant mode of scattered light is elastic Rayleigh scattering, a small proportion (1 to 10^9^ or 10^10^) of the photons is scattered inelastically. These photons shift to a higher or lower energy status resulting in stokes and anti-stokes scattering [1].

Raman spectra provides both qualitative and quantitative molecular-level information. The basis of the qualitative information is the fingerprint nature of the Raman shift, which is unique to each material. This makes Raman spectroscopy also usable in an aqueous environment [2], and an interesting and suitable technique for ophthalmic purposes. Raman spectroscopy is a non-contact and non-destructive technique with real-time visualization, which make it also suitable for *in vivo* application.

Biological samples often emit fluorescence signals that may interfere with Raman signals since the intensity of the fluorescence emission has a much higher yield than Raman signals [3]. Further, hardware related artefacts (instrumental noise) are found in Raman spectra. In order to extract Raman signal from the raw acquired spectrum, it is therefore necessary to pre-process the acquired spectra [4]. As recognized by Byrne et al. no standardized protocols are available for this purpose yet [5]. Hence, we developed a method to deal with multiple source background influences. This paper guides you through the steps taken to optimize Raman spectra and make them ready for analysis as done in the study from Bertens et al. [6]. For the full data-set of this project we refer to the supplementary data of Zhang et al. [7].

## Background of the data processing

**Fig. 1:**
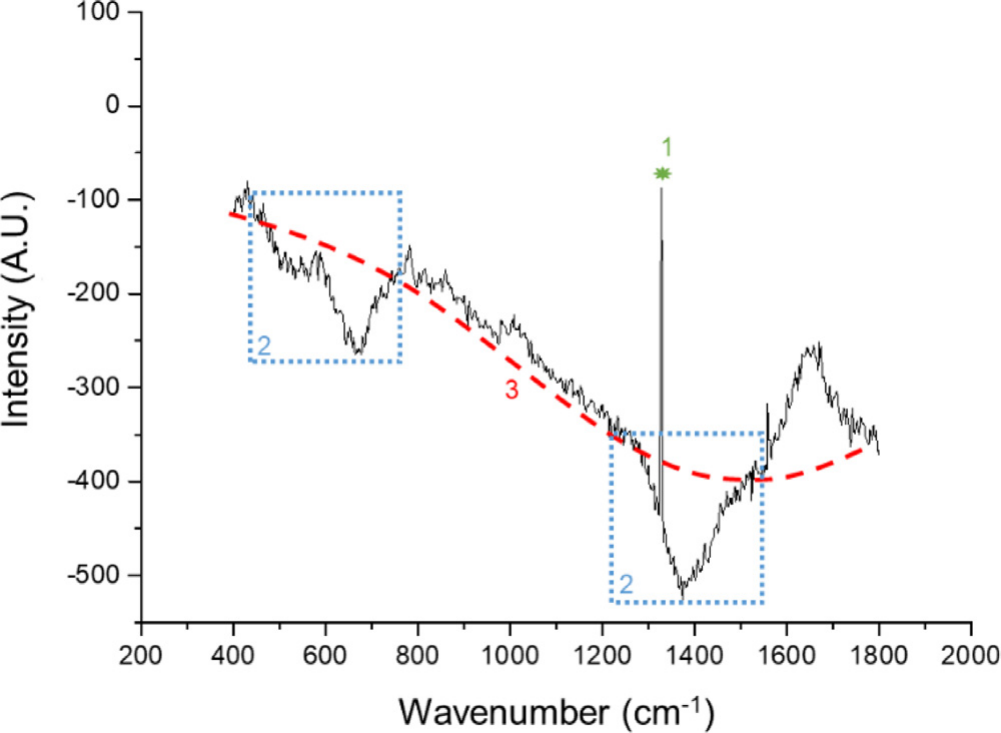
Example of a Raman spectrum. (1, green) Shows a cosmic ray spike, (2, blue) shows instrumental influences on the spectrum, and (3, red) shows a 5^th^ degree polynomial fitting for background correction.

**Fig. 2:**
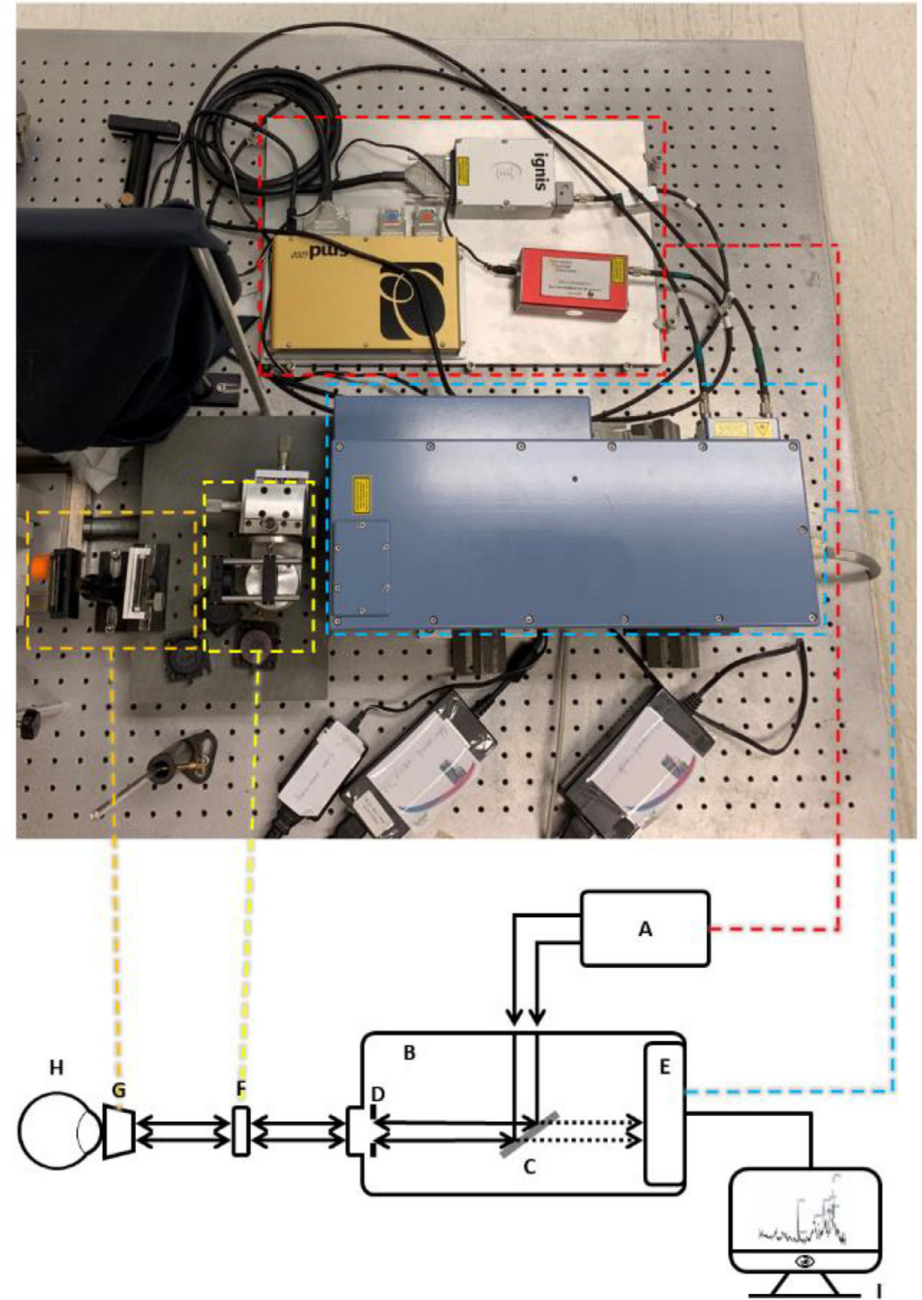
Raman set-up. (A) laser (red dashed region); (B) Raman module (blue dashed region), with (C) filter for Raman scattered light, (D) 25 µm pinhole and (E) integrated charge-coupled device (CCD); (F) collimation f80 lens (yellow dashed region); (G) f60 lens with a Gonio (one-mirror) lens, or objective (Jena lens), or a f80 lens (orange dashed region); (H) sample; and (I) computer (didn't show in the photograph). Arrows indicate direction of (backscattered) laser light; dashed arrows indicate direction of Raman-Scattered light.

**Fig. 3:**
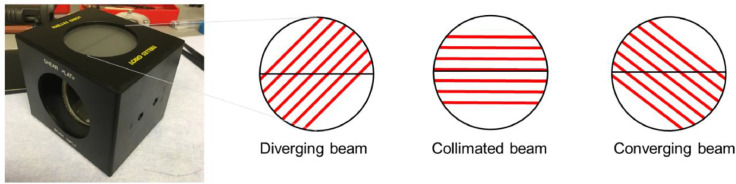
Melles Griot shear plate and the patterns it provides with different types of emitted light.

As mentioned earlier, there is no gold standard for the processing of Raman data. Several approaches have been proposed to minimize the influence from background fluorescence [5]. Raman scattering is an instantaneous effect, whereas fluorescence requires time to occur. If one can switch on and off the detector (or a filter) at a high temporal resolution, fluorescence signal could be prevented from interfering with the Raman signal. However, this is expensive, complicated, and commercially not available [8,9]. Therefore, the most accepted method for fluorescence background subtraction is polynomial fitting, for which unfortunately no standardized protocols are available (Fig. 1, Fig. 2, Fig. 3) [5]. Zhao et al. introduced an automated polynomial background subtraction method for biomedical applications, which could subtract the background [10]. Zhang et al. also developed a proper automated method for fluorescence background subtraction named: “automatic pre-processing method for Raman imaging data set (APRI)” [11]. However, both methods encountered difficulties when handling spectrums containing instrumental noise. In some *in vivo* experiments, the contribution from instrumental noise is inevitable and cannot be neglected, thus affecting the conventional polynomial methods. Hence, further treatments have been developed to eliminate the instrumental noise. Perez-Pueyo et al. introduced a morphology-based baseline removal method for Raman spectrums [12]. It employs Tophat filtering using basic operations as dilation and erosion to filter the features beyond or below a pre-set threshold, thereby removing the instrumental noise (Fig. 1, Fig. 2).

A third influencer affecting Raman spectra are cosmic rays. Cosmic rays create spikes that are randomly generated due to cosmic radiation (Fig. 1-1). Cosmic rays affected different wavenumbers each time they occur, and can easily be detected by comparing different frames of one measurement. Spikes created by cosmic rays need to be removed before the frames are averaged, else they can be interpreted as peaks [4,11].

## Materials and methods

### Materials

• Power conditioner: ONEAC PCm750I, 220–240 V, 3.1 A, 50/60 Hz
• Laser with wavelength 785 nm: Laser Model SM 785 nm purchased from Innovative Photonic Solutions; Output Power 50 mW; Bandwidth 9.73 GHz.
• Laser with wavelength 671 nm: Laser model: Ignis 671, purchased from Laser quantum; Output Power 100 mW; Bandwidth 30 GHz.
• Spectrometer: Model HPRM 2500, produced by River Diagnostics International BV. Specifications:
  ○ Spectral region coverage: 350 cm^−1^ - 1800 cm^−1^ with the 785 nm laser and ~2500 cm^−1^ - 4000 cm^−1^ with the 671 nm laser.
  ○ Spectral resolution: 2 cm^−1^ throughout the spectral region
  ○ Pinhole size: 25 µm
  ○ Back-illuminated deep-depletion CCD-camera: with 1024 × 128 pixels, air-cooled to −60 °C. Camera control software included.
• Computer: HP Compaq 6200 Pro-Microtower with operation system Windows^Ⓡ^ 7 Pro-OA.
• Jena lens: Planachromat LD 25x/0.5 ∞/0(2)-A, focus length is 10.1 mm.
• Mirror: Beam steering mirror assembly, model G063713000.
• Melles Griot Shear-plate
• Fibres: Diamond^Ⓡ^ FC APC/PM 20,853,190,002 for 850 nm and FC APC/PM 20,871,100,001 for 630 nm
• GonioLens, Haag-Streit Meridian; CGA1
• Edmund Optics lenses: f60 (60 mm focus point), f80 (80 mm focus point)

### Set-up of the Raman system

A modular confocal Raman spectroscopic system was used in the study. The Raman system was connected via a power conditioner, to prevent power peaks to disturb the measurements and to protect the system. The Raman system is equipped with a diode-emitting laser of 785 nm with a continuous power of 26 mW, and a 671 nm diode- emitting laser with a continuous power of 14 mW. Raman spectra were recorded with a high-performance Raman module model 2500 with a charge-coupled device (CCD) operating at −60 °C. This module introduces the laser light through a diamond optical fiber, shapes and conditions the beam through a pinhole to the measurement stage (Fig. 2). The emitting light from the spectrometer is collimated using a converging lens (f80 see Fig. 2-f). Collimation of the light was checked using the Melles Griot shear-plate. The lens was moved along the laser optic axis towards or away from the exit aperture of the spectrometer until the stripes provide a collimated position (Fig. 3).

**Fig. 4:**
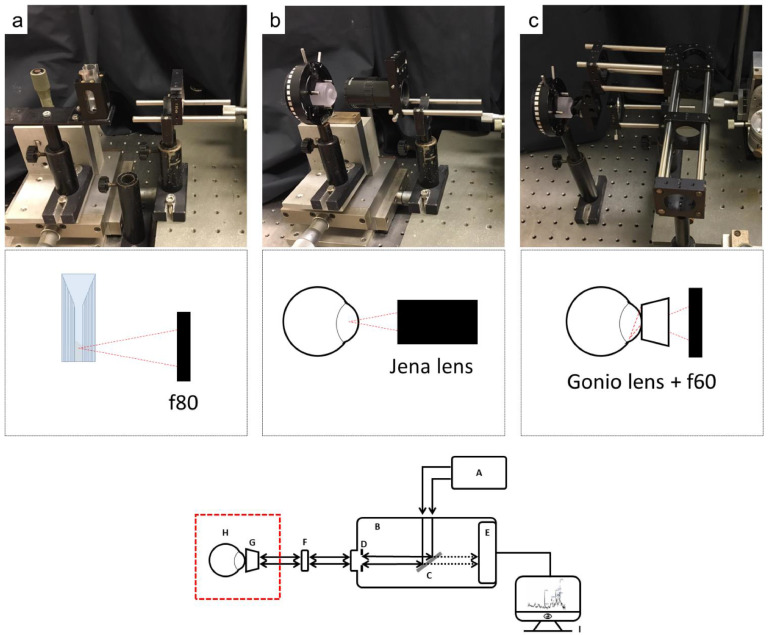
Details of the focus area of the Raman system (red dashed line in the scheme). (a) The set-up for cuvettes using a f80 lens, (b) Jena lens for focus in the anterior chamber of an eye, and (c) the Gonio lens in combination with a f60 focus lens for focus in the anterior chamber of an eye. The red dashed triangles show the focus position of the set-up.
• Jena lens set-up (Fig. 4b)
  • In front of the sample, a long-working-distance microscope objective lens, Jena lens.
• Gonio lens set-up (Fig. 4c)
  • In front of the sample, first a lens with a f60 lens is placed, followed by a Gonio lens. The Gonio lens was connected to the cornea of an eye (*in vivo* or *ex vivo*) using topically applied Methocel® 2%.

Three types of sample set-ups were performed:

• Cuvette set-up (Fig. 4a)
  • In front of the sample, a f80 lens was used when the sample was measured in a Brand® cuvette.

### Calibration

When the laser from the Raman system is collimated, the lens used for the measurement is set in place and the system is calibrated by built-in calibration procedure of the spectrometer. Hereafter, the system is further calibrated by the reference spectrum obtained by the provided National Institute of Standards and Technology (NIST)-standard calibration glass (was provided with the spectrometer). The full calibration was done according to the spectrometer manual. All measurements were performed in the dark.

### Positioning

**Fig. 5:**
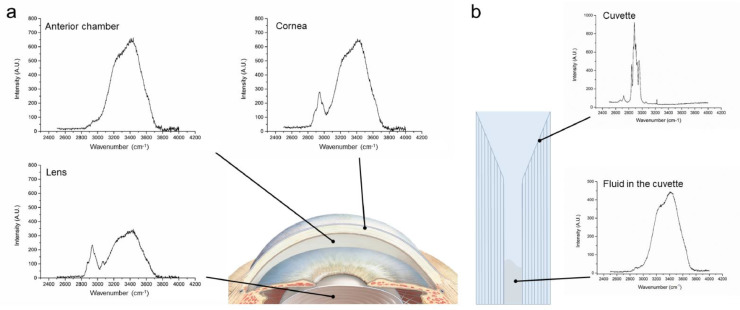
Location determination using high wave number measurement (671 nm Laser). (a) in the eye, and in (b) a cuvette.

The location in the sample was determined using the 671 nm laser, to create a high wave number signal (Fig. 5). In the eye, the cornea provides a protein peak (2800 cm^−1^– 3000 cm^−1^) followed by a water band (3000 cm^−1^–3800 cm^−1^). The anterior chamber only has a water band (3000 cm^−1^–3800 cm^−1^), and the lens has an extra protein peak around 3100 cm^−1^ besides the protein peak located at 2800 cm^−1^–3000 cm^−1^ and a water peak at 3000 cm^−1^–3800 cm^−1^. (b) In a cuvette, when focussed on the cuvette multiple high-intensity signals occur (between 2000 cm^−1^–3000 cm^−1^). When focussed on the fluid in the cuvette a water peak occurs (3000 cm^−1^–3800 cm^−1^).

### Data acquisition

**Fig. 6:**
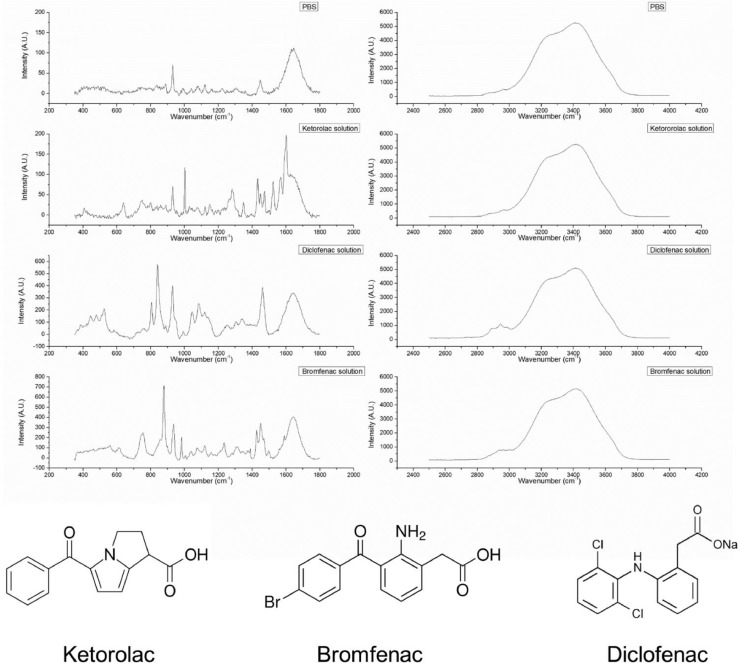
Raman spectra providing fingerprint signal (left column) and a high wave number signal (right column) of PBS and three different drugs (ketorolac (Acular^Ⓡ^), Bromfenac (Yellox^Ⓡ^), and Diclofenac (Naclof^Ⓡ^)) in ophthalmic solution. With corresponding molecular structure.

When the laser was correctly positioned, fingerprint-signal of the material was measured with the 785 nm laser and exported as ‘*.txt*’ file further processing. An example of a measurement is provided in Fig. 6.

## Data processing

### Removal of cosmic ray spikes

All Raman spectra were loaded into OriginPro 9.0.0 (64 bit ed. OriginLab corp. Northampton, US) and were one-by-one checked (manually) for cosmic ray spikes. The wavenumbers affected by cosmic ray spikes were replaced by the values of the same wavenumbers from another frame. When this was done, the files were saved and loaded into MatLab^Ⓒ^ (Version 2017b, The Mathworks Inc., Natick, MA, US) for further processing.

### Averaging of the frames, and removal of background- and instrumental noise

The following process is programmed in the MatLab^Ⓒ^ file (“*Polynomial_Tophat_background_subtraction _methods.m*”), provided with the manuscript.

**Fig. 7:**
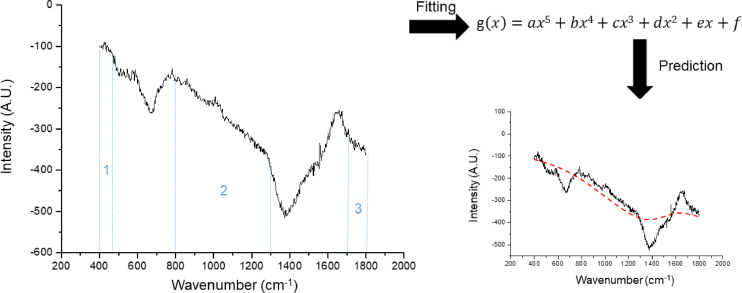
Overview of partial polynomial fitting. The spect rum is divided into different zones (1, 2, and 3), where after, a line was fitted through those zones based on a 5^th^ order polynomial function. The predicted line was withdrawn from the graph.

**Fig. 8:**
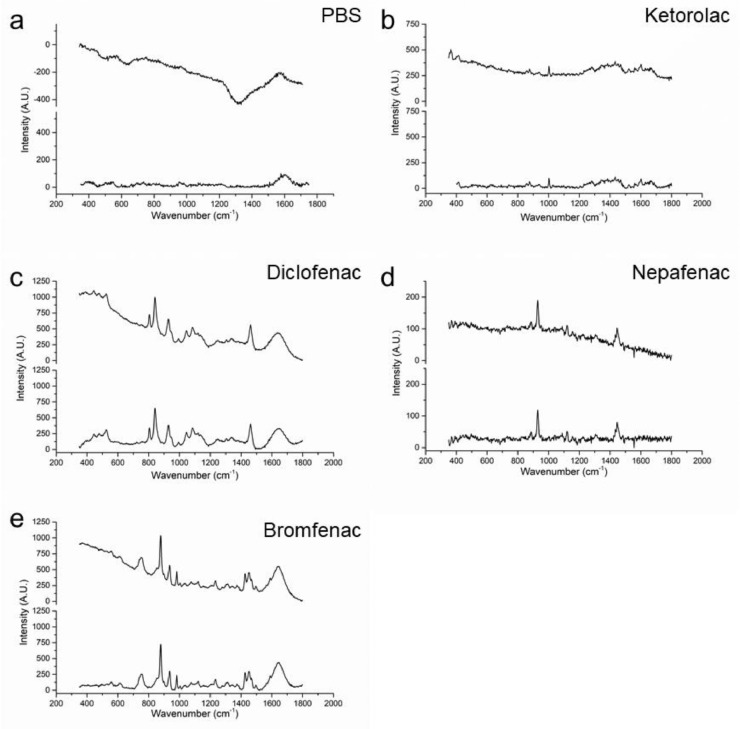
Effect of the processing on the data. (a) PBS in a rabbit eye, (b) ketorolac in a porcine eye, (c) diclofenac in a porcine eye, (d) nepafenac in a porcine eye, and (e) bromfenac in a porcine eye. The upper line shows RAW Raman signal and the lower line represents a processed Raman signal. Exposure time 30 s, average of 3 frames.

First, frames were averaged to reduce fluctuations. Because the baseline has a strong influence on the polynomial approximation, the polynomial degree must be selected according to the shape of the baseline. In our system, using eyes, a 5^th^ degree polynomial fitting resulted in the most optimal background correction (figure S1). Therefore, we applied partial 5^th^ degree polynomial fitting with the morphology approach of Perez-Pueyo et al. [12]. to remove instrumental noise. First, all spectra were dissected in different zones, 350 cm^−1^ to 450 cm^−1^, 450 cm^−1^ to 750 cm^−1^, 750 cm^−1^ to 1250 cm^−1^, 1250 cm^−1^ to 1650 cm^−1^, and 1650 cm^−1^ to 1800 cm^−1^. Zones that only contain fluorescence (400 cm^−1^ to 450 cm^−1^, 800 cm^−1^ to 1200 cm^−1^, and 1600 cm^−1^ to 1800 cm^−1^)(Fig. 7, zone 1, 2, and 3) are used calculate the polynomial function coefficients. The zone containing the water-peak (1550 cm^−1^ to 1650 cm^−1^) was excluded from the polynomial function fitting calculation. The achieved 5^th^ degree polynomial function was applied on the full spectrum (400 cm^−1^ to 1700 cm^−1^) to remove the fluorescence background (Fig. 7). Hereafter, the morphology-based Tophat method from Perez-Pueyo et al. [12]. was applied to eliminate instrumental noise. Examples of processed Raman signals are shown in Fig. 8.

**Fig. 9:**
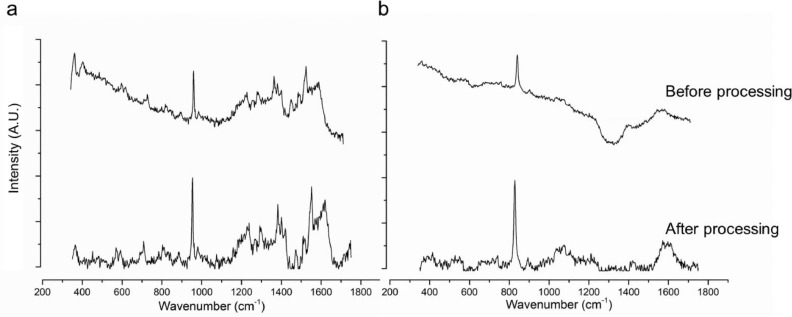
Effect of the processing on instrumental influences. (a) Shows a graph without instrumental influences (PBS) and (b) shows a graph with instrumental influences (rabbit eye). The upper line shows a RAW Raman spectrum and the lower line represents a processed Raman spectrum. Sample (a) is an *ex vivo* porcine eye, measured with Gonio lens, treated with 1.25 % ketorolac tromethamine (ophthalmic solution), exposure time 60 s, 3 frames. Sample (b) is an *in vivo* measurement of a rabbit eye (non-treated), measured with Gonio lens, exposure time 30 s, 2 frames.

Fig. 9 shows the effect of data processing using the MatLab^Ⓒ^ program on a sample without (Fig. 9a) and with (Fig. 9b) instrumental noise. In both occasions, a flat baseline is observed, and in Fig. 9b instrumental noise is reduced without affecting the peaks. A full overview of the corrected data can be found in Bertens et al. [6]. and the full data-set is available supplementary to the manuscript from Zhang et al. [7].

## Appendix Supplementary materials

Image, application 1
