# Supplementary material for: Pipeline for the removal of hardware related artifacts and background noise for Raman spectroscopy
Source: MethodsX. 2020 Apr 21;7:100883. doi: 10.1016/j.mex.2020.100883 (PMC7200319; doi:10.1016/j.mex.2020.100883)
Supplement: Supplementary file 1 [file mmc1.docx]

| 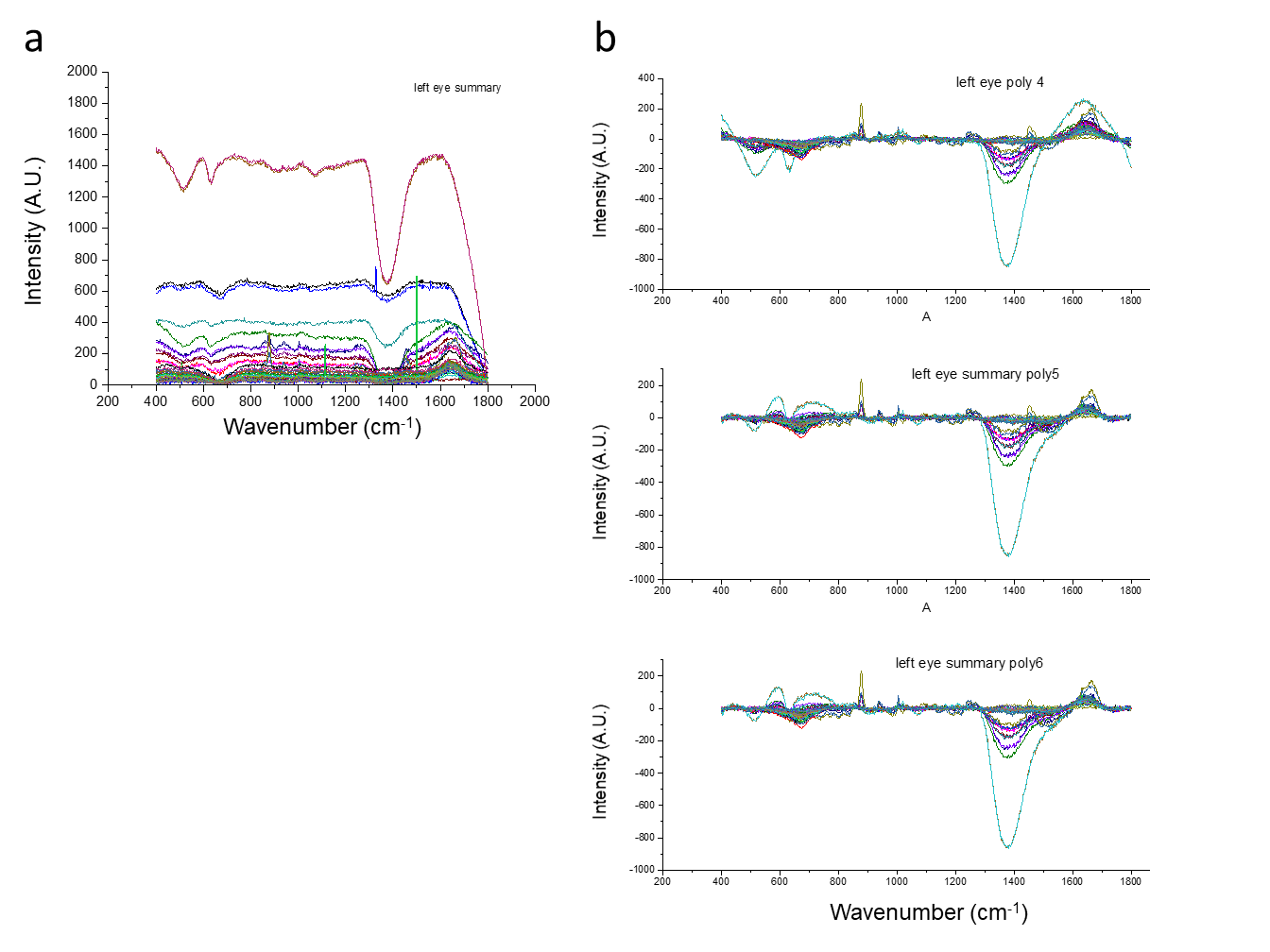 |
| --- |
| **Figure S1.** Optimization of the polynomial fitting. (a) RAW data before polynomial correction. (b) Data corrected using a 4^th^, a 5^th^, and a 6^th^ polynomial fitting function as shown in figure 5. No difference between the 5^th^ and 6^th^ polynomial fitting was observed; therefore, 5^th^ order polynomial fitting was used during the corrections. |
